# Major Depressive Disorder and Measures of Cellular Aging: An Integrative Review

**DOI:** 10.1155/2013/469070

**Published:** 2013-04-14

**Authors:** Patricia Anne Kinser, Debra E. Lyon

**Affiliations:** Department of Family and Community Health Nursing, School of Nursing, Virginia Commonwealth University, P.O. Box 980567, Richmond, VA 23298-0567, USA

## Abstract

Major depressive disorder (MDD) affects millions of individuals and causes significant suffering worldwide. It has been speculated that MDD is associated with accelerated aging-related biological and functional decline. To examine the accelerated aging hypothesis, one of the biomarkers under study is leukocyte telomeres, and specifically the measure of telomere length and telomerase activity. This review integrates findings from eleven human studies which evaluated telomere length and telomerase activity, in order to synthesize the state of the current science and to inform the development of new knowledge and enhance nursing research of depression using appropriate biobehavioral measures. Although preliminary, the findings from this integrated review suggest that there is evidence to support a conceptualization of depression as a stress-related condition in which telomeres shorten over time in relation to cumulative exposure to the chronic stress of depression. For the purposes of testing in future nursing research, visual representations of the theoretical connection between stress vulnerabilities, depression, and health outcomes and key moderators and mediators involved in this conceptualization are provided. The findings from this review and the conceptual framework provided may be a useful step towards advancing therapeutic nursing interventions for this debilitating chronic condition.

## 1. Introduction

Major depressive disorder (MDD) affects millions of individuals and causes significant suffering worldwide. The lifetime prevalence of MDD is approximately 16.2% [1] with women experiencing a disproportionately higher burden of MDD than men [2, 3]. The current DSM-IV-TR diagnostic criteria for MDD include a depressed mood and/or anhedonia for at least two weeks, plus additional symptoms such as excessive worrying, guilt, suicidal ideations, psychomotor changes, and alterations in sleep, weight, appetite, and cognitions [4]. Up to 50% of depressed individuals experience inadequate symptom relief from typical pharmacologic treatments [5]. Those with partial or no responses to treatment experience significant decreases in quality of life and functionality [6]. In addition to prolonged psychological distress, individuals with major mental illnesses, such as MDD, have shorter life expectancies and higher rates of other chronic medical conditions, such as cardiovascular disease, metabolic disorders, and chronic pain conditions [7, 8] compared with the general population. Thus, it has been speculated that MDD is associated with accelerated aging-related biological and functional decline. One of the biomarkers under study to examine the accelerated aging hypothesis is leukocyte telomeres and specifically the measure of telomere length and telomerase activity. This review integrates findings from human studies using biomarkers of telomere length and telomerase activity, in order to synthesize the state of the current science and to inform the development of new knowledge and enhance nursing research of depression using appropriate biobehavioral measures.

### 1.1. Potential Mechanism of MDD: Psychological Stress and Cellular Aging

Multiple potential mechanisms have been indicated in the etiology of MDD, many of which highlight the role of stress in both the development and the potential outcome of living with MDD. Psychological life stress and MDD are most often comorbid and there is a cyclic relationship in neurological mechanisms involved in mood and stress responsivity. For example, there is a “kindling” effect in the chronic nature of depression, such that every major depressive episode (MDE) increases the likelihood of recurrence [9]. Stress and depression are clearly linked, as depression may be a cause and/or outcome of chronic stress and increased stress levels can significantly affect the duration and degree of symptoms of depression [10–13]. Stress is considered to be one of the most significant predictors of health and MDD is a leading contributor to disease and a major cause of disability in the United States [14–16]. Stress and depression are associated with increased morbidity and mortality [10, 17], and this may be due to the fact that individuals with MDD have a significantly increased risk of other chronic medical illnesses, such as cardiovascular disease, diabetes, osteoporosis, and overall frailty/disability, [18–20]. This correlation between MDD and aging-related illnesses suggests that MDD may be related to accelerated cellular aging [21]. 

The connection between MDD and accelerated cellular aging may be via inflammatory pathways, and thus inflammatory states are of particular interest in depression research for several reasons. First, inflammation-mediated “sickness behaviors” (e.g., social withdrawal, anhedonia, fatigue) are classic symptoms of depression [9, 22]. Second, inflammation has been linked to telomere shortening and inflammation is linked to many conditions of aging, such as cardiovascular disease, endocrine disorders, and dementia [21, 23]; cytokines, which are proteins involved in intercellular signaling for the regulation of injury and infection, may be responsible for this link. Proinflammatory cytokines, such as IL-6 and TNF-alpha, have been demonstrated to increase telomerase activity in human multiple myeloma cells and peripheral blood lymphocytes [24–26]. Other studies have demonstrated significant reductions in telomerase activity in relation to the inflammatory milieu of acute stress or exposure to high levels of cortisol [27–29]. Third, inflammation causes oxidative stress, which in turn leads to telomere shortening [30]. For these reasons, the association of MDD with inflammation is a target of keen research interest. 

### 1.2. Telomere Measures

The measure of TL is a novel approach for understanding correlations between genetic or environmental factors and physical and mental health. As “caps” on the ends of chromosomes, telomeres are protein complexes which protect the chromosome during cell division. Telomeres become shorter with every cell division, eventually limiting the cell's lifespan. Telomerase is an enzyme which maintains telomere length by adding DNA base pairs (bp) to the shortened telomere, thus promoting longevity of the cell [31]. Compelling evidence from recent research suggests that one mechanism by which stress adversely affects physical health may be related to decreased activity of the enzyme telomerase which results in shortened telomere length (TL) [32–35]. When telomerase activity (TA) decreases, telomeres shorten faster than normal, which can lead to loss of cell function or even cell death [36]. Considered to be an indicator of accelerated aging, shortened TL and decreased TA are associated with adverse health sequalae, such as increased overall mortality and morbidity and increased rates of cardiovascular events, cerebrovascular events, cancer, reduced fertility, pulmonary fibrosis, and dementia, [35, 37–42]. Affected by dynamic changes in telomerase activity, TL may be a novel outcome measure of the cumulative effects of various cellular stressors, both genetic and environmental [31]. Changes in telomere length may take months or years to be detectable in research studies, whereas changes in telomerase activity may be seen over much shorter periods [28]. Telomerase activity may be influenced by nonmodifiable factors, such as age and genetic vulnerabilities, and modifiable factors including chronic stress, oxidative stress/inflammation, infections, and elevated cortisol, [27, 31, 36, 42, 43]. It has been theorized that increased TA may initially occur in immune cells as a compensatory response for extensive stress-induced shortening of telomeres and that eventually telomerase activity decreases to subprime levels, leading to continued accelerated aging [26].

## 2. Materials and Methods

**Figure 1:**
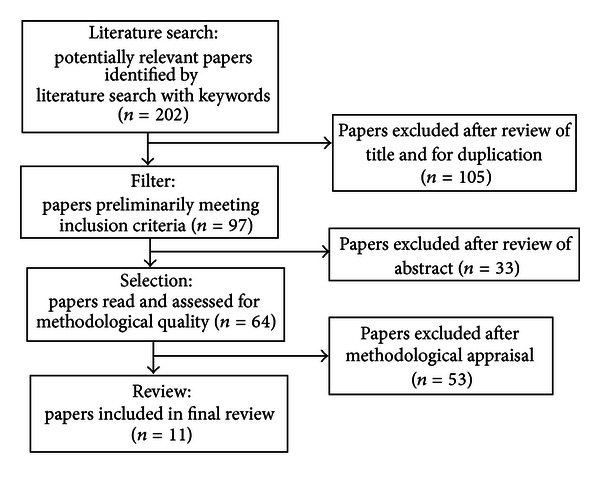
Flow diagram of the literature selection process.

An integrative review was used to examine human research studies that focused on MDD and measures of cellular aging, telomere length, and levels of circulating telomerase. The search of the electronic databases PubMed/Medline and CINAHL from their date of inception to October 2012 used key words “depressive disorder,” “telomere,” “telomerase,” “telomere shortening,” “telomere homeostasis,” “epigenesis, genetic.” Hand searches were also completed using references from previously published studies and literature reviews. Articles that were retrieved via these multiple search methods (*n* = 202) were reviewed for duplication and for whether they met the following inclusion criteria: (1) research study with human participants; (2) participants met diagnostic criteria for major depressive disorder (MDD) and/or a major depressive episode (MDE); (3) the study used quantitative outcome measures of telomere length (TL) or telomerase activity (TA); (4) the study reported clear methodologies for measuring telomere length. Because MDD often presents as multiple recurrent major depressive episodes (MDEs), we included studies of participants reporting MDEs. In addition, many individuals with depressive symptoms do not always seek care and may not have received an official diagnosis of MDD or MDE [1]; we also evaluated studies that included participants self-reporting depressive symptoms.  Figure 1 represents the process of literature selection. A total of 11 articles met these inclusion criteria and are included in this review.  Table 1 is provided as a summary of the findings.

## 3. Results 

 A summary of findings related to the association of depression with TL and/or TA may be found in Table 1. Eleven studies evaluated these measures in participants with MDD and/or MDEs or self-reported depression. The majority of the studies are cross-sectional in design, with only one longitudinal study of participants with MDD. In the earlier studies (2006–2010), the Southern blot method was used for measuring telomere length. However, most of the studies were published since 2011 and used the quantitative fluorescence in situ hybridization (qFISH) or quantitative polymerase chain reaction (qPCR) methodologies for measuring telomere length, suggesting that interest in telomere biology and the state of the science is rapidly progressing. Potentially complicating our analysis of the review findings, the types of participants evaluated in the studies are quite varied; for example, one study evaluated outpatients with heart disease and MDD [44], another evaluated unmedicated outpatients with MDD [18], and another evaluated hospitalized patients with MDD starting antidepressant medication [45]. The primary constant among all of the studies was that participants had either a diagnosis of MDD (8 studies) or a previous history of MDEs/depressive symptoms (3 studies) and telomere length was measured (all studies).

### 3.1. Studies of Participants with MDD

 Eight studies evaluated the association between telomere length and major depressive disorder. All of the studies with the exception of one found that participants with MDD had significantly or close to significantly shortened telomeres compared to controls. Two of the three studies that measured inflammation found a significant relationship between proinflammatory markers (CRP, IL-6) and shortened telomeres in depressed participants. To provide a chronological perspective of the studies and the development of the science over time, each study is briefly reviewed, in chronological order, including a discussion of key findings and limitations. 

Simon and colleagues (2006) [46] evaluated telomere length in participants with either MDD (*n* = 15), bipolar disorder with comorbid anxiety (*n* = 15), or bipolar disorder without comorbid anxiety (*n* = 14), as compared to age-matched controls (*n* = 44). Leukocyte telomere length data was analyzed using the Southern blot method. There were no TL differences when comparing the three types of mood disorders, so the groups were combined for comparison with age-matched controls. Telomere length was significantly shorter in those with mood disorders compared to the control group, with an overall mean difference of 660 base pairs (*t* = 3.16, *P* = 0.002). This significance remained when adjusting for age, gender, and a lifetime smoking history. Simon et al. suggest that this mean difference reflects approximately 10 years of accelerated aging in those with a mood disorder. Neither age nor smoking status was predictive of telomere length. One limitation in the reporting of this study is that it was unclear whether the age-matched controls were closely evaluated to rule out mood disorders. Like many of the other studies of this nature, this study was limited by a lack of ethnic/racial diversity, no BMI data (which is known to affect TL), and no evaluation of stress levels.

 Hartmann and colleagues (2010) [45] conducted an evaluation of the telomere length of hospitalized patients with MDD (*n* = 54) who were receiving various levels of treatment: low doses of antidepressants (*n* = 20), high doses of antidepressants (*n* = 16), and antidepressants plus electroconvulsive therapy (*n* = 18). Each of these groups were compared to each other and to healthy age-matched controls (*n* = 20). The mean leukocyte telomere length (analyzed by Southern blot) of all of the depressed subjects (7.20 ± 0.61 kb) was significantly shorter than the TL of the control subjects (7.55 ± 0.54 kb; *P* < 0.01); this reflects a mean difference of approximately 350 base pairs. Interestingly, there was no correlation noted between TL and smoking status, gender, duration or severity of depression, or number of hospitalizations. However, consistent with other studies, all subjects had age-dependent shortening of the telomeres (approximately 20 base pairs per year) no matter whether they were healthy or had depression. This data is limited in that BMI data was not reported, 100% of the subjects were Caucasian, and there was no evaluation of stress levels.

 Similarly, findings from the study by Wolkowitz and colleagues (2011) [18] suggest that chronicity is a key concept in the relationship between depression and telomere length. In this study of unmedicated individuals with MDD (*n* = 18) as compared to healthy age, sex, and ethnicity-matched controls (*n* = 17), those who had experienced depression the longest had significantly shorter telomeres than the control group (*F* = 2.87, *P* = 0.05), which corresponded to approximately 280 base pairs or 7 years of accelerated aging. Although there was no overall difference between groups in terms of telomere length or measures of oxidative stress and inflammation (IL-6 and F2-isoprostanes and Vitamin C levels), IL-6 concentrations were inversely correlated with telomere length in the depressed group (*F* = 3.29, *P* < 0.05) and was approaching significance among all participants (*F* = 2.45, *P* = 0.07). Wolkowitz and colleagues (2012) [49] continued this line of research in a study of patients completing an open-label study of sertraline (*n* = 16) compared to healthy matched controls (*n* = 18). Baseline telomerase activity levels were elevated in those with MDD as compared to the controls (*P* = 0.007) and, similarly, higher baseline telomerase activity was positively correlated with higher levels of depression and stress (*P* < 0.05). Lower baseline telomerase activity was correlated with improved depression severity over time. Surprisingly, there were no significant relationships noted between telomerase activity and telomere length, nor were there correlations between telomerase activity and measures of inflammation (IL-6, CRP).

 Hoen et al. (2011) [44] evaluated a group of older predominantly male patients with coronary heart disease and found that those with MDD (*n* = 206) had shorter mean telomere lengths (0.86 ± 0.02 versus 0.90 ± 0.01, *P* = 0.02) than those without depression (*n* = 746). This difference in TL reflects approximately 2.3 years of accelerated aging in those with depression. The finding became a trend (*P* = 0.06) when controlling for characteristics that differed between groups (age, gender, BMI, smoking status, physical inactivity, antidepressant use, anxiety, left ventricular ejection fraction, and statin use). A five-year follow-up found that, although depression seemed to initially be correlated with shorter telomeres, depression status did not predict the subsequent shorter telomere lengths. However, this finding is complicated by the fact that the researchers did not evaluate depressive symptoms or diagnoses at followup, so it is unknown whether this lack of correlation is meaningful. 

Lung and colleagues (2007) [47] evaluated 253 outpatients with MDD, compared to healthy controls (*n* = 411) for differences in telomere length. The most significant finding was that the group with MDD had shorter telomeres than the control group (8.17 ± 0.61 and 9.13 ± 1.49, resp., *t* = 11.64, *P* < 0.01), although potential confounders were not measured (life stress, BMI, smoking, physical activity, and others). Similarly, Wikgren and colleagues (2012) [50] found that outpatients with recurrent MDD (*n* = 91) had significantly shorter leukocyte TL than healthy controls (*n* = 451), adjusting for age and gender (*P* = 0.001; approximately 277 base pair difference). Interestingly, telomere length was not correlated with smoking status, BMI, or C-reactive protein levels in either group. Testing the hypothesis that long-term chronic stress leads to a hypoactive HPA axis and that depression is a state of chronic stress, Wikgren et al. assessed participants' stress with the objective measure of a low-dose dexamethasone suppression test (DST). Of the participants with MDD, those with low post-DST cortisol levels had shorter TL (*P* = 0.007, difference of 332 base pairs), suggesting that TL differences between MDD and healthy participants may be related to hypocortisolemia.

The only study that did not find a correlation between depression and telomere length was the study by Malan et al. (2011) [48] which evaluated the relationship between the development of MDD and leukocyte telomere length in women who were survivors of rape, hypothesizing that those with shorter TL would be more susceptible to the development of MDD or PTSD. Of the 64 women in the study, 36% (*n* = 23) had MDD at baseline and 41% (*n* = 31) had MDD at a 3-month follow-up appointment. There was no association between relative TL and the development of MDD and there was a nominally significant association between TL and PTSD. This study may be limited in that it evaluated a very specific population who had just recently experienced a traumatic event to test a unidirectional hypothesis that a shorter telomere length leads to depression. It has been suggested in the literature that the relationship between a mood disorder and telomere length is most likely bidirectional, in that a stressful depressive episode may actually lead to telomere changes and not vice versa [21]. Furthermore, depressive symptoms may not show up immediately in individuals recently experiencing a trauma. 

### 3.2. Studies of Participants with Self-Reported MDEs or Depression Symptoms

In order to understand whether self-reported depressive symptoms alone (without a clinical diagnosis of depression) are related to differences in telomere length, we have included studies that involved participants who provided self-reports of either previous depressive episodes or depressive symptoms. Of the three studies of this kind, one found a significant correlation between depressive episodes and telomere length. Elvsashagen and colleagues (2011) [51] evaluated the correlation between lifetime depressive episodes and PBMC telomere length (measured with quantitative fluorescence in situ hybridization qFISH) in individuals with bipolar-II disorder (*n* = 28) as compared with healthy age, gender, and education-matched controls (*n* = 28); there was a positive correlation between the number of depressive episodes and the percentage of short telomeres (*r* = −0.35, *P* = 0.02) and a trend such that as the lifetime number of depressive episodes increased, the mean telomere length decreased by an average of 552 bps (*r* = −0.35, *P* = 0.08). This is an interesting finding which suggests that chronicity may be a key factor in the relationship between mood disorders and telomere length. Consistent with the concept of “kindling” (one episode of depression increases the likelihood of another), it appears that increased numbers of depressive episodes may enhance the shortening of telomeres. These findings are in line with the literature which suggests that accelerated aging occurs due to the physiologic and psychological stress induced by depressive episodes. 

 Two studies evaluated participants with self-reported depression symptoms only (no diagnosis of MDD or a MDE) and neither found statistically significant results. Shaffer et al. (2012) [52] sampled apparently healthy homogeneous Nova Scotians (*n* = 2225) and found that those with depressive symptoms did not have significantly shorter TL than those without, when adjusting for age and sex. It is important to note that, in this study, there was no clinical diagnosis of depression whereas the other studies reviewed here had some method of clinician-based assessments of a depressive disorder. Hoen et al. (2013) [53] conducted a sample of subjects in The Netherlands (*n* = 974) in which 9% had either MDD or dysthymia. This study found that the presence of a depressive disorder did not predict shorter telomere length at a two-year followup (*P* = 0.753). As with the Shaffer study, a limitation of this study was that the depression instrument was self-administered and not clinically confirmed. Another significant limitation of this study was that psychopathology and telomere length were measured at time points separated by multiple years and there was no repeated measure of psychopathology.

## 4. Discussion

The findings from this integrated review suggest that there is preliminary evidence to support a conceptualization of depression as a stress-related condition in which telomeres shorten over time in relation to cumulative exposure to the chronic stress of depression. The majority (8 of 11) of the studies reviewed herein found that individuals with MDD or those with a history of MDEs/depressive symptoms have significantly or close to significantly shortened telomeres compared to controls, reflecting an “accelerated aging” of approximately 2–10 years. In addition, preliminary findings suggest a relationship between depression, markers of inflammation (such as IL-6 and CRP), and telomere length. Finally, although telomerase is known to be involved in telomere maintenance, telomerase activity was only measured in one study so the significance of the findings with regards to depression is unclear.

The findings from this review are limited for a number of reasons. First, there have been relatively few studies focusing on major depression independent of other major chronic illnesses and there was a variety of definitions of depression used. Second, the majority of these are cross-sectional which limits the ability to determine a cause-effect relationship. Third, these studies have used a variety of measures so it is difficult to directly compare results. Fourth and finally, as reflected in Table 1, many of these studies have numerous limitations such as unclear reporting about screening/diagnosis of MDD, a lack of control for potentially confounding variables (e.g., stress, BMI, smoking, physical activity, anti-inflammatory medication use), homogenous samples or unclear reporting of ethnicities/races of samples, testing unidirectional hypotheses, lack of a clinical diagnosis of depression, and mismatched assessment time points of biomarkers and depression. 

Despite these limitations, the clinical implications of telomere shortening in depression warrant attention and future long-term studies. Although cross-sectional studies limit the ability to determine causality and very few longitudinal studies have been published at the time of this writing, telomere length may be a clinically important objective outcome measure for longitudinal intervention studies [21, 31, 33]. Individuals with stress and depression may have accelerated telomere shortening which has been seen in previous nonexperimental research on individuals with psychological stress and mood disorders. A cross-sectional study of healthy women by Epel and colleagues (2004) [29] revealed that the perception of high levels of stress and the chronic nature of stress is correlated with shortened TL. Evidence from a number of other epidemiologic studies suggests that in cases of chronic stress and/or clinical psychological disorders, telomerase activity decreases, thus resulting in shortened telomeres by anywhere from 240 to 1750 bp [32, 36, 46]. While objective measures of stress and clinically diagnosed mood disorders show correlations with shortened TL, subjective cognitive perceptions of life stress also appear to be significantly correlated with TL. High scores on the Perceived Stress Scale (PSS) were correlated with shortened TL in premenopausal women [34, 36]. Furthermore, participants' self-rating of depression level has been inversely correlated with TL, such that high levels of depression are seen in individuals with shorter TL [43]. 

Future research is warranted to determine the implications of changes in TL and TA over time. The combination of measuring TA and TL may be relevant for use in longitudinal studies because changes in TA may be seen within a few days, whereas TL may take months or years to change [54]. However, an inconsistent relationship between TA and TL has been demonstrated in studies of chronic stress. Findings from some studies suggest that TA is compensatorily induced by stress and telomere shortening whereas others suggest that TA is decreased in chronic stress or depression and thus TL is shortened (see [21, 54]). Future research should continue to account for the fact that TA and TL may be influenced by nonmodifiable factors (e.g., age, race/ethnicity, and gender) and, modifiable factors (e.g., stress, inflammation, infections, elevated cortisol, BMI, and physical activity) [21]. Age, race, and BMI are often used as covariates in studies which have evaluated stress levels and mental health [34–36, 47, 55]; however, the majority of the studies in this review did not do so. Research suggests that TL is inversely related to BMI and age [42, 56] and African Americans have longer TL than Caucasians [42]. There is a small dose effect of age on TL by decade before age 50 [31], where average TL shortening during adulthood may be 30–60 bp per year [29, 33]. It is unclear, however, whether TL should be used as a marker of illness vulnerability versus actual ongoing disease or rather as a reflection of the sequelae of stress involved in depression. 

**Figure 2:**
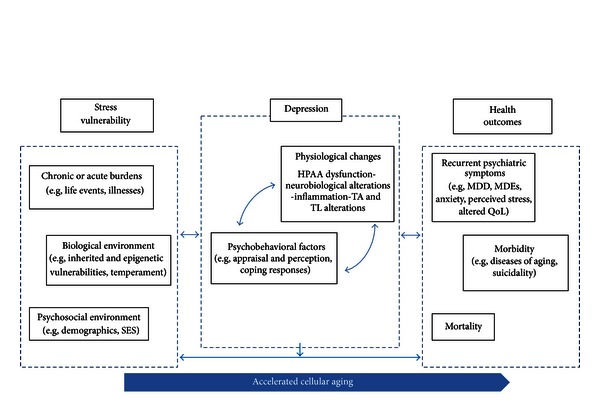
Accelerated cellular aging: stress vulnerability, depression, and health outcomes. SES = socioeconomic status; ECD = early childhood development; HPAA = hypothalamus-pituitary-adrenal axis; TA = telomerase activity; TL = telomere length; MDD = major depressive disorder; MDEs = major  depressive episodes; QoL = quality of life.

**Figure 3:**
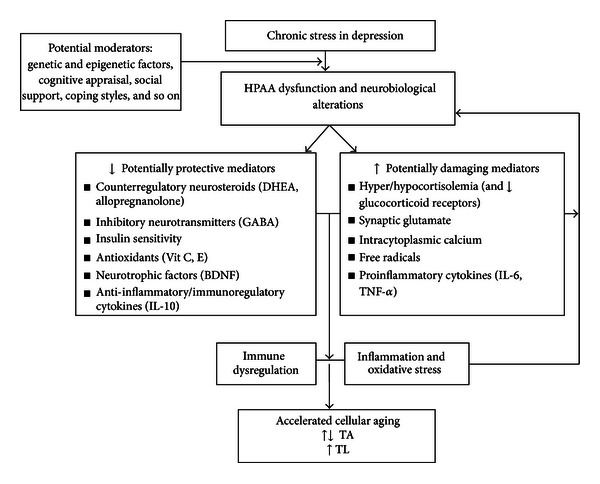
Highly simplified schema of mediators and moderators potentially related to cell damage or dysfunction, accelerated cellular aging, and depression.

Considering the extant literature supporting the connection between psychosocial stress, depression, and telomere length (see reviews such as [21, 54, 57]), it is clear that there are interrelated pathways involved in stress and depression.  Figure 2 provides a visual representation of theoretical connections between stress vulnerabilities, depression, and health outcomes, in which the physiological changes occurring in depression may be reflected in biomarkers of accelerated cellular aging [9, 21, 54, 57]. For example, stress hormones and inflammation may be important mediators between telomere length, depression, and stress; a recent study found that high levels of pessimism, typical in depression, may be correlated with shortened leukocyte telomere length and elevated levels of IL-6 [58].  Figure 3 provides an oversimplified schema of potential moderators and mediators involved in accelerated cellular aging (for more details, see [59]). These models do not depict the complex relationships between many of these mediators but rather provide preliminary theoretical models for testing in future research. For instance, although shortened telomere length may reflect an accumulation of stress with chronic depression, it may be too early to confidently assert that TL may be used as a reliable biomarker in depression and research is needed to determine whether telomeric aging is reversible [60]. These models may be tested and applied in order to enhance our understanding of underlying mechanisms of interventions for depression and stress; for example, recent studies suggest that mindfulness, yoga, and relaxation techniques may be therapeutic interventions for mitigating the effects of chronic stress and depression, possibly preventing stress-related accelerated cellular aging [33, 55, 61–63]. 

## 5. Conclusion

In conclusion, we have conducted an integrated review of eleven research studies which evaluated the association of depression with telomere length. The findings suggest that there is preliminary evidence to support the conceptualization of depression as a state of stress-induced accelerated cellular aging, as represented by shortened telomeres. Despite the multiple limitations of the various studies and considering that it is still unclear how well telomere length may serve as a biomarker in depression, the findings are consistent with the literature which suggests telomere pathology is relevant in other chronic illnesses and chronic stress. It has been suggested that telomere shortening may be the “missing link” for understanding morbidity and mortality in depression [57]. Although the state of the science may still be relatively new in this field, TL appears to be an important novel outcome measure to incorporate into longitudinal studies. For the purposes of testing in future research, we have presented a visual representation of the theoretical connection between stress vulnerabilities, depression, and health outcomes (Figure 2) as well as a simplified schema of key moderators and mediators involved in this conceptualization (Figure 3). Nursing research has historically been highly involved in investigating underlying mechanisms of and interventions for depression. The provocative findings from this review provide insights into the possible relationships between depression, chronic stress, and cellular aging and this may be a useful step towards advancing therapeutic nursing interventions for this debilitating chronic condition. 

## Figures and Tables

**Table 1:** Overview of studies: telomere length and depression.

First author, date	Type of study, total *n*	Method	TL shorter in participants with MDD or MDE or depression symptoms	Mean difference in TL, approx. years of accelerated aging	Other findings	Study limitations
Studies of participants with MDD						

Simon, 2006 [46]	Cross-section, *n* = 88	LTL-Southern blot	Yes*	660 bps, ~10 yrs	—	a, b, c
Lung, 2007 [47]	Cross-section, *n* = 664	LTL-Southern blot	Yes*	96 bps, ~2 yrs	—	a, b, c
Hartmann, 2010 [45]	Cross-section, *n* = 74	LTL-Southern blot	Yes*	350 bps, ~5 yrs	—	a, b, c
Hoen, 2011 [44]	Cross-section, *n* = 952	PBMCs-qPCR	Yes**	97 bps, ~2 yrs	—	c
Wolkowitz, 2011 [18]	Cross-section, *n* = 35	LTL-qPCR	Yes*	280, ~7 yrs	IL6 inversely correlated with TL in depressed pts*	b
Malan, 2011 [48]	Cross-section, *n* = 64	LTL-qPCR	BTL not associated with development of MDD in 3 months	—	TL associated with development of PTSD**	b, d
Wolkowitz, 2012 [49]	Longitudinal, *n* = 34	PBMCs TRAPeze assay for TA	Yes* (reported in Wolkowitz 2011 [18])	280, ~7 yrs	BTA elevated in depression* and high stress*; lower BTA predicted better response to antidepressant*; no correlation between TA, IL6, CRP, and TL	—
Wikgren, 2012 [50]	Cross-section, *n* = 542	LTL-qPCR	Yes*	277 bps, ~4 yrs	TL shorter and CRP higher in MDD with low post-DST cortisol levels*	a, c

Studies of participants with history of MDEs or self-reported depression symptoms						

Elvsashagen, 2011 [51]	Cross-section, *n* = 56	PBMCs-qFISH	Yes**	552 bps, ~9 yrs	—	b, c, e
Shaffer, 2012 [52]	Cross-section, *n* = 2225	LTL-qPCR	No	—	—	a, b, c, e
Hoen, 2013 [53]	Longitudinal, *n* = 974	LTL-qPCR	No	—	Anxiety disorder associated with shorter TL over time**	e, f

*Significant: *P* < 0.05; **trend: *P* < 0.10.

LTL: leukocyte telomere length; BTL: baseline telomere length; BTA: baseline telomerase activity; DST: dexamethasone suppression test; qPCR: quantitative polymerase chain reaction; qFISH: quantitative fluorescence in situ hybridization.

^a^Unclear if controls actually screened for MDD or controls answered in negative regarding previous diagnosis of MDD.

^b^Factors potentially affecting TL not evaluated/reported (stress, BMI, smoking status, physical activity, anti-inflammatory medications).

^c^Homogeneous group or ethnicity/races unreported.

^d^Testing unidirectional hypothesis (telomere length leads to development of depression).

^e^No clinical diagnosis of depression (self-report of previous MDE or depression symptoms).

^f^Biomarkers measured at different time point than depression diagnosis.
